# Are the Unified Protocols for Transdiagnostic Treatment of Emotional Disorders in Children and Adolescents as Effective for OCD as for Anxiety and Depression?

**DOI:** 10.3390/children12040529

**Published:** 2025-04-21

**Authors:** Lauren Milgram, Madison E. Bigler, Elizabeth R. Halliday, Kiara R. Timpano, Jill Ehrenreich-May

**Affiliations:** Department of Psychology, University of Miami, Coral Gables, FL 33146, USA

**Keywords:** OCD, anxiety, depression, unified protocol, cognitive behavioral therapy, transdiagnostic, youth

## Abstract

**Background**: Obsessive–compulsive disorder (OCD) in youth commonly co-occurs with other affective disorders (e.g., anxiety, depression). Exposure and response prevention (ERP) is the front-line treatment for OCD but may require significant adaptation to treat co-occurring symptoms or complex comorbidity patterns. Preliminary evidence suggests that the Unified Protocols for Transdiagnostic Treatment of Emotional Disorders in Children and Adolescents (UP-C/A) are effective in reducing OCD symptoms. Still, it is not yet known if the UP-C/A are comparably effective to treat OCD as they are for anxiety and depression, the disorders for which there is the most robust empirical support. **Methods**: This study compared trajectories of OCD, anxiety, and depression symptom change among 388 youth who received UP-C/A treatment (*M* = 15 sessions) at a university-based research clinic. We also examined whether youth with (*n* = 60) and without (*n* = 328) an OCD diagnosis demonstrated comparable improvements in anxiety, depression, and transdiagnostic treatment targets (i.e., anxiety sensitivity, cognitive flexibility, and distress tolerance). **Results**: OCD symptoms improved at a slower rate than anxiety and depression symptoms during the first half of UP-C/A treatment but at a comparable rate to anxiety and depression symptoms during the second half of treatment. Youth with and without OCD exhibited comparable improvements in anxiety, depression, anxiety sensitivity, cognitive flexibility, and distress tolerance across the treatment course. **Conclusions**: Findings support the efficacy of UP-C/A treatment for co-occurring OCD and affective disorders but suggest that initiating exposure earlier in the treatment course may confer additional benefits.

## 1. Introduction

Obsessive–compulsive disorder (OCD) is a common mental health condition characterized by intrusive thoughts, urges, or images (obsessions) and behaviors intended to alleviate the distress associated with the obsessions (compulsions). OCD is associated with significant personal distress and functional impairment [1,2]. While OCD may develop at any time throughout the lifespan, OCD is most likely to onset during childhood and adolescence [3,4]. OCD in youth is associated with a range of adverse outcomes, including familial distress [5], social difficulties [6], academic impairment [7], and decreased quality of life [8]. Developing, testing, and disseminating effective treatments is critical to alleviate suffering and improve outcomes for youth with OCD.

Exposure and response prevention (ERP), a type of cognitive behavioral therapy (CBT), is the front-line psychosocial treatment for OCD [9]. Broadly, exposure therapy techniques entail planned contact with feared situations or stimuli. For OCD, exposure typically involves engaging in thought exercises or activities that trigger an obsessive thought, while response prevention entails making concentrated effort to resist performing the desired mental or behavioral ritual that alleviates the distress associated with the thought. ERP is thought to elicit change in OCD symptoms by providing opportunities to experience a natural decrease in fear in the absence of the compulsive behavior (i.e., habituation) [10,11], to learn that a feared outcome does not occur in the presence of a fear stimulus (i.e., expectancy violation) [12,13], and/or to reduce sensitivity to uncomfortable physiological or emotional states (i.e., increased distress tolerance) [14,15]. A large body of research supports the effectiveness of ERP for OCD in youth [16,17,18,19,20]. Current estimates indicate that up to 70% of youth in clinical trials of ERP exhibit treatment response [21,22]. Still, only a minority of youth exhibit full symptom remission after a standard course of ERP alone, and up to 10% of youth exhibit chronic and enduring symptoms that do not respond to ERP [23]. Long-term outcome data further indicate that even those who do exhibit response or remission initially often experience a re-emergence of symptoms later in life [24]. There is a growing interest in examining factors that moderate treatment response to inform efforts to improve treatment outcomes.

One factor that consistently moderates response to ERP for OCD is the presence of other co-occurring psychiatric disorders. Many studies have found that youth who present with co-occurring psychiatric disorders exhibit poorer rates of response to ERP for OCD [25,26,27]. These findings are particularly concerning given that comorbidity is the norm rather than the exception for youth with OCD. Current estimates indicate that up to 80% of youth with OCD present with at least one additional psychiatric disorder [24,28]. OCD most frequently presents alongside affective disorders such as anxiety and depressive disorders [29,30,31], although co-occurring neurodevelopmental disorders (e.g., attention deficit hyperactivity disorder, tic disorders, autism spectrum disorder) are also common [32,33,34]. While the hypothesized mechanism through which comorbidity impacts treatment response varies by co-occurring disorder, researchers have generally theorized that the presence of co-occurring psychiatric disorders may pull focus from OCD treatment and require clinicians to stray from ERP-specific strategies [27]. Clinical practice guidelines generally suggest focusing on OCD if it is the primary presenting concern and addressing comorbidities if they interfere with ERP; yet, practicing clinicians often report greater reluctance and/or difficulty to use exposure-focused strategies with clients who present with multiple comorbid psychiatric disorders [35,36]. The presence of comorbid affective disorders, in particular, can impact youth clients’ motivation for OCD treatment and ability to engage in exposure exercises necessary to elicit symptom improvement [25].

Transdiagnostic CBT approaches offer an alternative to single domain (i.e., disorder- or domain-specific) approaches such as ERP for OCD. Transdiagnostic therapies focus less on disorder-specific content and more on general CBT strategies that can aid a range of presenting symptoms. Transdiagnostic CBT approaches were developed in response to evidence that many mental health concerns share core dysfunctions and that, by targeting key mechanisms, multiple psychiatric concerns can be treated concurrently in an efficient manner and without deviating from adherence to a single intervention [37,38,39]. A recent randomized trial indicated that transdiagnostic CBT elicited equal symptom improvement and less attrition compared to single domain CBT for anxiety or depression alone [40]. Moreover, some evidence suggests that transdiagnostic CBT approaches yield greater client and clinician satisfaction than single domain approaches [41,42]. Although research on transdiagnostic CBT approaches for OCD is more limited, these approaches are hypothesized to be beneficial given the high rates of comorbid affective disorders among youth with OCD, as well as evidence that OCD and co-occurring affective disorders share many symptom features and core dysfunctions [43,44,45]. In particular, a growing body of literature indicates that deficits in emotion regulation processes, including dysfunctions in anxiety sensitivity, cognitive flexibility, and distress tolerance, act as maintenance factors for OCD and other affective disorders [14,46,47,48]. A transdiagnostic CBT approach that leverages ERP principles in addition to other CBT techniques to address these broader core dysfunctions may provide benefits above and beyond stand-alone ERP for youth with OCD and co-occurring affective disorders.

The Unified Protocols for Transdiagnostic Treatment of Emotional Disorders in Children and Adolescents (UP-C/A) [49] are transdiagnostic CBT treatments for children and adolescents, respectively, that target the core dysfunctions in anxiety sensitivity, cognitive flexibility, and distress tolerance that maintain a range of youth affective disorders. The UP-C/A incorporate a variety of CBT techniques that are presented in a modular format such that clinicians may determine the optimal order and presentation of content based on the client’s presenting symptoms. When delivered sequentially by module, approximately the first half of UP-C/A treatment consists of psychoeducation, behavioral activation, and cognitive restructuring, and approximately the second half of treatment consists of mindfulness techniques (e.g., present-moment awareness, non-judgmental awareness) and exposure exercises (e.g., “generalized emotion exposure”, in vivo exposure). Several studies support the efficacy and effectiveness of the UP-C/A for anxiety and depressive disorders [50,51,52,53,54,55,56], and researchers are increasingly evaluating the use of the UP-C/A to treat other affective and emotion-related disorders (e.g., eating disorders, misophonia) [57,58,59]. Furthermore, emerging evidence indicates that the UP-C/A elicits changes in its intended transdiagnostic mechanisms central to the maintenance of affective disorders [60]. Despite these promising findings for affective disorders broadly, little research has directly examined the effects of the UP-C/A specifically for youth with OCD, who may differ in their response compared to youth with anxiety and depressive disorders.

A preliminary study conducted by our team [61] examined change in OCD symptoms over time among youth with affective disorders (including but not limited to OCD) receiving UP-C/A treatment. Findings from the study indicated that, on average, youth self-reported and parent-reported OCD symptoms decreased significantly and steadily over the course of UP-C/A treatment. The rate of OCD symptom improvement was not moderated by youth age or gender. Further, youth treatment engagement and satisfaction did not differ between youth with and without an OCD diagnosis. While these results are promising, the preliminary study did not examine whether the amount or rate of change in OCD symptoms is comparable to or different from the amount and rate of change in anxiety and depression, the disorders for which there is the most robust empirical support for the UP-C/A. More research is needed to understand whether the UP-C/A produces similar changes in OCD symptoms as in anxiety and depressive symptoms and whether the UP-C/A can effectively target its intended mechanisms (i.e., anxiety sensitivity, cognitive flexibility, and distress tolerance) among youth with OCD.

The present study examined whether treatment using the UP-C/A is equally effective for OCD as it is for anxiety and depressive disorders. First, we compared trajectories of OCD, anxiety, and depression symptom change during UP-C/A treatment (Aim 1). We examined trajectories across the first half of UP-C/A treatment and second half of UP-C/A treatment separately given that, if delivered sequentially by module, the first half of UP-C/A treatment presents a broader range of skills applicable to a range of affective disorders, and the second half of UP-C/A treatment focuses more explicitly on exposure, which is likely to be more targeted toward OCD symptoms. We hypothesized that OCD symptoms would improve during both the first and second halves of treatment but would exhibit greater relative improvements during the second half of treatment. We hypothesized that the trajectories of OCD symptoms would be comparable to the trajectories of anxiety and depression symptoms. Second, we examined whether trajectories of anxiety and depression symptoms differ between youth with and without an OCD diagnosis (Aim 2). We hypothesized that youth with OCD would exhibit comparable improvements in anxiety and depression symptoms to youth without OCD. Finally, we examined whether the UP-C/A are equally effective at targeting core dysfunctions in anxiety sensitivity (Aim 3a), cognitive flexibility (Aim 3b), and distress tolerance (Aim 3c) among youth with and without an OCD diagnosis. We hypothesized that youth with OCD would exhibit comparable improvements in anxiety sensitivity, cognitive flexibility, and distress tolerance to youth without OCD.

## 2. Method

### 2.1. Study Design

Youth participants were referred to the university-based research clinic by their parents or primary caregivers. Youth and families seeking treatment for emotional concerns completed informed consent and assent and participated in a semi-structured psycho-diagnostic interview with a graduate student or post-doctoral clinician to assess presenting symptoms and treatment eligibility. Diagnostic interviews were conducted using the Anxiety Disorders Interview Schedule for DSM-IV, Child and Parent Versions (ADIS-IV-C/P) [62,63], the Anxiety Disorders Interview Schedule for DSM-5, Child and Parent Versions (ADIS-5-C/P) [64], or the Mini-International Neuropsychiatric Interview for Children and Adolescents (MINI-KID) [65,66] depending on differences in clinic procedures at the time of youth enrollment. Clinical severity ratings were assigned by the clinician for each presenting diagnosis on a scale from 0 (i.e., no symptoms) to 8 (i.e., extreme symptoms). Clinical severity ratings of 4 (i.e., moderate symptoms with distress and impairment) or above signified clinically significant symptoms warranting a diagnosis. Clinicians were trained to reliability on the interview measures by completing three assessments with trained clinicians and “matching” on diagnoses and clinical severity ratings. Each diagnostic assessment was reviewed by a clinical supervisor during weekly supervision meetings. Youth who were determined to be eligible for treatment (see Section 2.2.1) were added to the clinic waitlist and, if still interested at the time of treatment availability, began treatment using the UP-C/A. Youth and parents completed questionnaires at pre-treatment (i.e., during the initial eligibility evaluation), mid-treatment (i.e., at approximately week 8 of the treatment course), and post-treatment (i.e., whenever participants completed treatment, which typically occurred around week 15 or 16 but varied by participant). Youth and families were compensated for their completion of the questionnaires.

### 2.2. Participants

#### 2.2.1. Inclusion and Exclusion Criteria

Participants included children and adolescents who presented with their parents or primary caregivers to a university-based research clinic in southern Florida, United States between May 2014 and June 2022 for treatment for emotional concerns (e.g., anxiety, depression, OCD and related disorders, irritability, and trauma and stressor-related disorders). Participants were determined to be eligible for treatment at the research clinic if they presented with a primary affective or emotion-related disorder of moderate severity that was deemed appropriate for weekly outpatient treatment using the UP-C/A. Participants were determined to be ineligible for treatment at the research clinic if they presented with a primary non-affective disorder requiring treatment or if they presented with psychotic symptoms, intellectual disability, or current suicidal or homicidal ideation requiring a higher or different level of care. Participants were also determined to be ineligible for treatment at the research clinic if there was no parent or caregiver able and willing to participate in treatment with the youth participant or if the family could not complete treatment in English or Spanish. An initial sample of 844 youth completed a diagnostic interview to assess treatment eligibility. Of these, 444 youth (52.6%) were determined to be eligible based on presenting with a primary affective or emotion-related disorder of moderate severity and were enrolled in UP-C/A treatment. Ineligible participants included 126 youth (14.9%) who were enrolled in other, non-UP-C/A clinical services within the clinic (e.g., trauma-focused CBT, cognitive behavioral intervention for tics), 157 youth (18.6%) who were referred out to external clinical services (e.g., in need of a higher level of care, non-affective disorder requiring care), and 117 youth (13.9%) who opted not to initiate treatment services (e.g., sought services elsewhere, no longer seeking treatment). Of the 444 youth who were determined eligible and enrolled in UP-C/A treatment, 56 youth (12.6%) were excluded from the present study because they fell below the age cut-off (i.e., younger than 8 years old) for the focal measure in this study, the Revised Children’s Anxiety and Depression Scale (see Section 2.3).

#### 2.2.2. Final Sample

The final sample of participants included 388 children and adolescents (51.5% female; 90.9% White; 70.8% Hispanic/Latino/a) between the ages of 8 and 18 years old (*M* = 12.5, *SD* = 2.9) and their parents or primary caregivers (86.2% mothers). The median annual family income was USD 100,000 (*M* = USD 139,051; *SD* = USD 153,972).

Most youth (90.2%) were diagnosed with more than one presenting disorder, and the average number of diagnoses among the sample was 2.9 (*SD* = 1.2). Almost all youth were diagnosed with a primary or co-occurring anxiety disorder (96.4%), while a smaller proportion of youth were diagnosed with a primary or co-occurring depressive disorder (35.8%). Sixty youth (15.5%) were diagnosed with primary (*n* = 33) or co-occurring (*n* = 27) OCD, and an additional twenty-six youth (6.7%) reported subclinical OCD symptoms that did not meet the threshold for diagnosis. Youth also often presented with co-occurring neurodevelopmental disorders (e.g., attention deficit hyperactivity disorder, autism spectrum disorder), trauma and stressor-related disorders (e.g., post-traumatic stress disorder), obsessive–compulsive-related disorders (e.g., excoriation), and externalizing disorders (e.g., oppositional defiant disorder).

Approximately equal numbers of youth received UP-C (51.3%) and UP-A (48.7%) treatment. Most youth were enrolled in individual treatment (78.9%), while a smaller number of youth were enrolled in group treatment (21.1%). Most participants received treatment services in person (55.2%), although a considerable minority of participants received telehealth (30.2%) or hybrid in-person/telehealth (14.7%) services. The average number of treatment sessions attended was 15.0 (*SD* = 7.5). See Table 1 for descriptive statistics for the full sample and split by OCD diagnostic status.

### 2.3. Measures

#### 2.3.1. Demographic Information

Demographic information, including youth age, biological sex, race, ethnicity, and annual family income, was collected from parents or primary caregivers at the pre-treatment diagnostic assessment.

#### 2.3.2. Anxiety, Depression, and OCD Symptoms

Revised Child Anxiety and Depression Scale (RCADS), Child and Parent Reports [67]: The RCADS-Child and RCADS-Parent are two parallel measures of emotional symptoms completed by youth (ages 8 and above) and parents, respectively. The RCADS includes 47 items assessing symptoms of common anxiety disorders (i.e., generalized anxiety disorder, social anxiety disorder, separation anxiety disorder, and panic disorder), depression, and OCD. Items are rated based on symptom frequency on a Likert-type scale from 0 (“Never”) to 3 (“Always”). A total symptom score can be calculated by summing all items. The RCADS also offers a T-scoring system to obtain symptom subscale and total scores normed by youth age/grade and biological sex. T-scores of 65 and higher indicate mild clinical elevation, and T-scores of 70 and higher indicate significant clinical elevation. The RCADS has demonstrated strong internal consistency and convergent and discriminant validity in previous studies [68,69]. In this study, RCADS T-scores for the generalized anxiety disorder, depression, and OCD symptom subscales were used to examine symptom trajectories over time. RCADS youth self-report and parent-report symptom subscales exhibited good internal consistency across the treatment course (α ranged from 0.74 to 0.92 across timepoints, reporters, and subscales).

#### 2.3.3. Transdiagnostic Factors

Childhood Anxiety Sensitivity Index (CASI) [70]: The CASI is an 18-item youth self-report measure of anxiety sensitivity, defined as the fear of anxiety-related symptoms due to the belief that these symptoms will lead to harmful physical, psychological, or social consequences [71]. CASI items are rated on a Likert-type scale from 1 (“None”) to 3 (“A lot”). Items are summed to create a total anxiety sensitivity score ranging from 18 to 54, and higher scores indicate greater anxiety sensitivity. The CASI has demonstrated strong convergent and discriminant validity in previous studies [72]. In this study, the CASI exhibited strong internal consistency across the treatment course (α ranged from 0.89 to 0.93 across timepoints).

Emotion Regulation Questionnaire for Children and Adolescents (ERQ-CA) [73]: The ERQ-CA is a 10-item youth self-report measure of two facets of emotion regulation: cognitive flexibility/reappraisal and emotion suppression. Cognitive flexibility is conceptualized as the ability to shift or reduce negative thoughts to reduce negative emotional states, whereas emotion suppression is conceptualized as the tendency to inhibit or conceal negative thoughts to temporarily avoid negative emotional states. ERQ-CA items are rated on a Likert-type scale from 1 (“Strongly disagree”) to 5 (“Strongly agree”). Items within each subscale are summed for a total score of cognitive flexibility/reappraisal, ranging from 6 to 30, and a total score of emotion suppression, ranging from 4 to 20. The ERQ-CA has demonstrated strong convergent and discriminant validity in previous studies [73,74]. For this study, only the cognitive flexibility/reappraisal subscale was used to align with study aims. Higher scores on this subscale indicate higher cognitive flexibility. The ERQ-CA cognitive flexibility/reappraisal subscale items exhibited strong internal consistency in this sample (α ranged from 0.83 to 0.85 across timepoints).

Distress Tolerance Scale (DTS) [75]: The DTS is a 15-item youth self-report measure of distress tolerance, which is defined as the ability to tolerate negative situations and emotional states. Items are rated on a Likert-type scale from 1 (“Strongly agree”) to 5 (“Strongly disagree”). Items are summed to yield four subscale scores (i.e., tolerability/aversiveness of distress [“tolerance”], appraisal of distress [“appraisal”], tendency for distress to disrupt functioning [“absorption”], and ability to regulate distress [“regulation”]) and one higher order factor score of global distress tolerance (GDT), which was used in the present study. The GDT is comprised of the 12 items of the tolerance, appraisal, and absorption subscales, and higher GDT scores indicate greater distress tolerance. The DTS full scale and GDT scale have demonstrated good internal consistency and sensitivity to treatment-related changes in youth samples [76,77]. In this study, the DTS-GDT scale demonstrated strong internal consistency (α ranged from 0.90 to 0.93 across timepoints).

### 2.4. Procedures

All procedures were conducted within an IRB-approved protocol.

#### 2.4.1. Treatment

Youth and their parents received transdiagnostic CBT using the Unified Protocols for Transdiagnostic Treatment of Emotional Disorders in Children and Adolescents (UP-C/A) [49]. The UP-C and UP-A are developmentally tailored adaptations of the adult Unified Protocol for Transdiagnostic Treatment of Emotional Disorders (UP) [78] for children (ages 5 to 12) and adolescents (ages 12 to 18), respectively, with affective disorders. The UP-C and UP-A focus on the same treatment components but vary slightly in structure and content to ensure developmental appropriateness for their respective age groups. While the UP-C and UP-A can both be delivered in individual or group format, the UP-C was originally developed to be delivered in group format and, compared to the UP-A, was more often delivered in group format in the present study. See Table 2 for descriptions of UP-C/A treatment content, theorized mechanisms of change, and the approximate treatment schedule. In addition to the youth-focused treatment content described in Table 2, the UP-C/A also include parental psychoeducation about children’s emotional symptoms and strategies to decrease accommodation of unhelpful behaviors, increase praise and rewards, reduce criticism, model healthy emotion regulation, and promote child independence. Parent-focused content can be delivered within either UP-C or UP-A but was more often and more explicitly a focus of UP-C treatment in the present study. Treatment using the UP-C/A is expected to span approximately 15 to 16 sessions (*M* = 15.0 sessions attended in this study). In the present study, treatment was delivered by graduate students and post-doctoral clinicians who received weekly supervision from licensed psychologists. The same iteration of the UP-C/A therapist guide was used across the duration of this study.

#### 2.4.2. Data Analysis

Missing data on demographics (e.g., age, sex) and treatment-related predictor variables (e.g., treatment format, number of treatment sessions attended) ranged from 0% to 7.0%. Missing data on pre-treatment questionnaires ranged from 14.9% to 20.6%. Missing data on pre-treatment questionnaires were not missing completely at random (Little’s MCAR Test *X*^2^(1296) = 1462.9, *p* = .001) but were associated with a range of demographic variables (i.e., younger age, greater likelihood of being male, greater likelihood of receiving UP-C compared to UP-A treatment, greater likelihood of receiving group compared to individual treatment, and greater likelihood of receiving treatment in hybrid/telehealth format). Missing data on pre-treatment questionnaires were determined to be missing at random (MAR) as these missing data could be explained by other variables present in the dataset [79]. Missing data on pre-treatment questionnaires and other predictor variables were handled with multiple imputation using Multivariate Imputation by Chained Equations from the “mice” package in RStudio Version 2024.12.1+563 [80] with *m* = 20 imputations. All demographic and treatment-related predictor variables were included as auxiliary variables in the imputation, consistent with current recommendations [81,82]. Missing data on outcome variables (i.e., repeated assessments of questionnaires during treatment) were handled using restricted maximum likelihood estimation.

We characterized the distributions of outcome measures (i.e., RCADS, CASI, ERQ-CA, and DTS) in terms of skewness and kurtosis and assessed the normality of model residuals with Q–Q plots. Pearson correlations were conducted to assess bivariate associations among all outcome measures. Multilevel modeling was conducted in RStudio using the lme4 package [83] to characterize trajectories of symptoms over time. To compare the trajectories of OCD, anxiety, and depression symptom change (Aim 1), multiple RCADS T-score symptom subscales (i.e., OCD, generalized anxiety, and depression) were nested within assessment timepoints (i.e., pre-treatment, mid-treatment, and post-treatment), which were nested within participants. Symptoms were modeled in a piecewise manner as to examine separate trajectories for (1) pre- to mid-treatment and (2) mid- to post-treatment. Predictors in this model were time, age, sex, race, ethnicity, family income (Z-scored for analysis), pre-treatment symptom severity (i.e., RCADS Total T-score), number of diagnoses, treatment format (individual or group), treatment location (in-person or telehealth/hybrid), number of treatment sessions attended, and symptom domain (i.e., OCD, anxiety, or depression). Treatment manual (UP-C or UP-A) was not included as a predictor in any model due to its high collinearity with youth age. The main effect of the symptom domain variable represented differences in severity of OCD, anxiety, and depression at pre-treatment. A time by symptom domain interaction term was included as the focal predictor to represent differences in trajectories of OCD, anxiety, and depression symptoms over time. We used planned orthogonal contrasts to compare trajectories between (1) OCD and anxiety/depression and (2) anxiety and depression. We examined simple slopes to describe trajectories for each of the three groups. To examine whether trajectories of anxiety and depression symptoms differed between youth with and without an OCD diagnosis (Aim 2), and to examine whether trajectories of anxiety sensitivity (Aim 3a), cognitive flexibility (Aim 3b), and distress tolerance (Aim 3c) differed between youth with and without an OCD diagnosis, we conducted separate, parallel multilevel models of change in these factors over time. OCD diagnosis (yes/no), the time by OCD diagnosis interaction, and covariates (i.e., age, sex, race, ethnicity, family income [Z-scored for analysis], pre-treatment RCADS symptom severity, number of diagnoses, treatment format, treatment location, and number of treatment sessions attended) were included as predictors in each model. We examined simple slopes to describe trajectories of symptoms for youth with and without an OCD diagnosis. For each model, we assessed multicollinearity by computing variance inflation factor (VIF) and tolerance (Tol) values, with VIF values above 5 and Tol values below 0.10 indicating potentially problematic levels of multicollinearity. We characterized effect sizes by computing Cohen’s *d* values for each predictor, with values between 0.2 and 0.5 signifying small effect sizes, values between 0.5 and 0.8 signifying medium effect sizes, and values above 0.8 signifying large effect sizes.

## 3. Results

### 3.1. Descriptive Statistics for Outcome Measures

**Figure 1:**
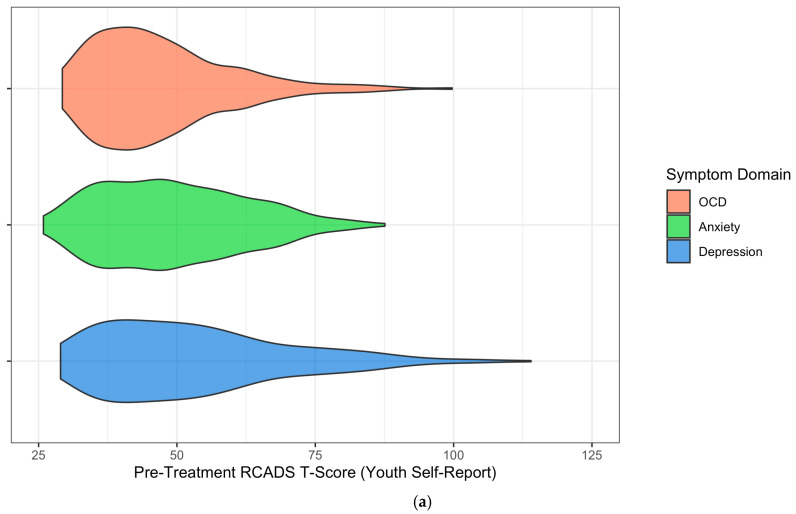

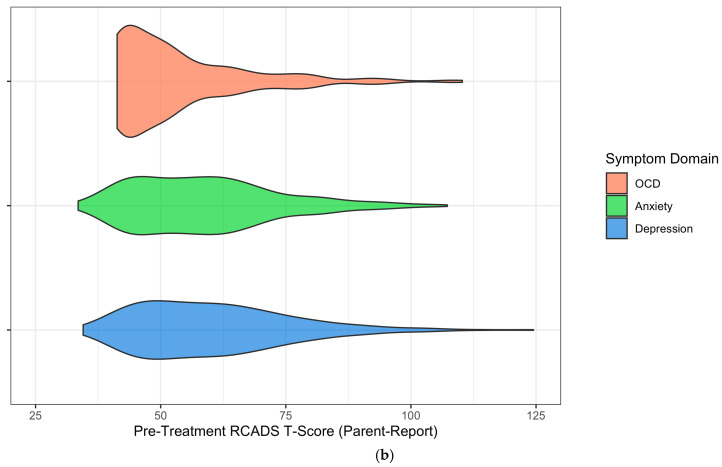
Distribution of symptoms (RCADS T-scores) by symptom domain at pre-treatment, based on (**a**) youth self-report and (**b**) parent-report. Note: RCADS = Revised Children’s Anxiety and Depression Scale. OCD = Obsessive–compulsive disorder.

Most outcome measures showed slight positive skew, suggesting that fewer youth and parents reported severe symptoms compared to those who reported low or moderate levels of symptoms. The mean pre-treatment RCADS Total T-scores for youth (*M* = 52.2, *SD* = 15.6) and parents (*M* = 62.4, *SD* = 14.6) indicated subclinical levels of affective symptoms within this sample. Youth self-reported low to moderate anxiety sensitivity (CASI *M* = 30.7, *SD* = 7.8), moderate cognitive flexibility (ERQ-CA *M* = 18.9, *SD* = 5.1), and moderate distress tolerance (DTS-GDT *M* = 35.3, *SD* = 11.0) at pre-treatment. Skewness and kurtosis values did not indicate major deviations from normality, and a visual inspection of Q–Q plots indicated that model residuals were approximately normally distributed. See Table 3 for skewness, kurtosis, and bivariate correlations among outcome measures at pre-treatment. See Figure 1a and 1b for comparisons of pre-treatment symptom distributions, indicating an overall lower endorsement of OCD symptoms compared to anxiety and depression symptoms in this sample.

### 3.2. Aim 1: Trajectories of OCD, Anxiety, and Depression Symptoms During UP-C/A Treatment

#### 3.2.1. Youth Self-Reported Symptom Trajectories

**Figure 2:**
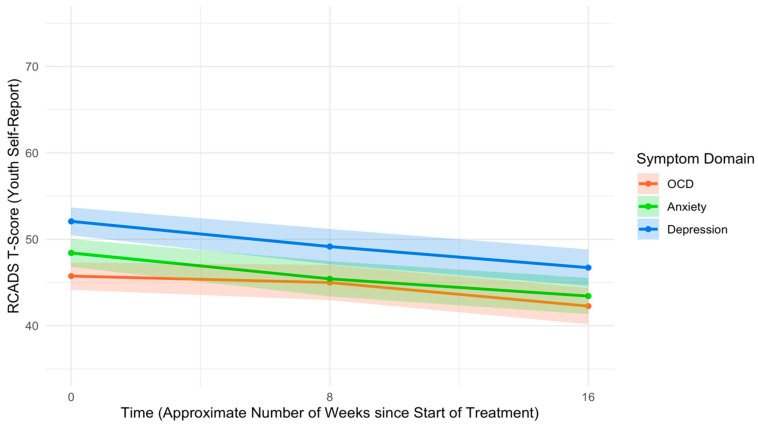
Trajectories of RCADS T-scores across treatment course by symptom domain, based on youth self-report. Note: RCADS = Revised Children’s Anxiety and Depression Scale. OCD = Obsessive–compulsive disorder.

An unconditional model examining change in youth self-reported RCADS Total T-scores indicated that affective symptoms improved significantly from pre- to mid-treatment (*B* = −0.41, *SE* = 0.13, *p* = .002, *d* = −0.36) and from mid-treatment to post-treatment (*B* = −0.38, *SE* = 0.15, *p* = .012, *d* = −0.29). Multilevel model results indicated that youth reported significantly higher anxiety/depression symptoms than OCD symptoms (*B* = 4.50, *SE* = 0.63, *p* < .001, *d* = 0.44) and significantly higher depression symptoms than anxiety symptoms (*B* = 3.65, *SE* = 0.73, *p* < .001, *d* = 0.31) at pre-treatment. Controlling for pre-treatment symptom severity and other covariates, OCD symptoms decreased at a slower rate than anxiety/depression symptoms from pre- to mid-treatment (*B* = −0.28, *SE* = 0.14, *p* = .046, *d* = −0.12). Simple slope analyses indicated that, while anxiety (*B* = −0.38, *SE* = 0.13, *p* = .004) and depression (*B* = −0.37, *SE* = 0.13, *p* = .005) symptoms both exhibited significant improvements from pre- to mid-treatment, OCD symptoms did not exhibit significant improvements during this time (*B* = −0.09, *SE* = 0.13, *p* = .466). On the contrary, there were no differences in rates of change between OCD and anxiety/depression from mid- to post-treatment (*B* = 0.06, *SE* = 0.16, *p* = .695, *d* = 0.02), nor were there differences in rates of change between anxiety and depression symptoms from mid- to post-treatment (*B* = −0.06, *SE* = 0.19, *p* = .768, *d* = −0.02). OCD symptoms improved significantly from mid- to post-treatment (*B* = −0.34, *SE* = 0.15, *p* = .025), and this rate of improvement was comparable to that of anxiety (*B* = −0.25, *SE* = 0.15, *p* = .101) and depression (*B* = −0.30, *SE* = 0.15, *p* = .046). See Table 4 for full multilevel model results, and see Figure 2 for trajectories of youth-reported symptoms across the UP-C/A treatment course.

#### 3.2.2. Parent-Reported Symptom Trajectories

**Figure 3:**
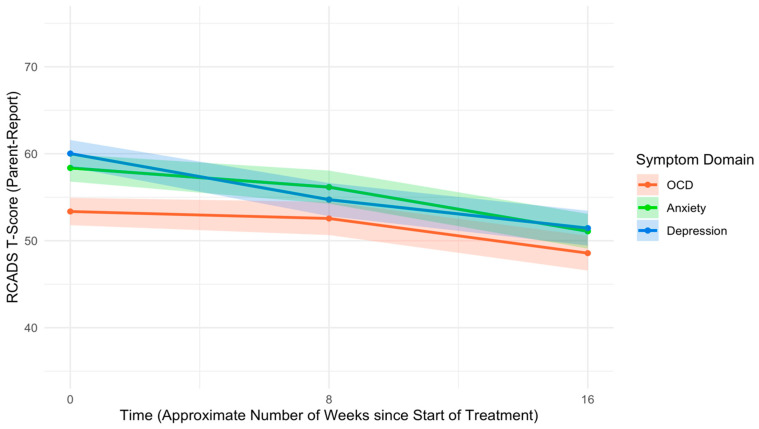
Trajectories of RCADS T-scores across treatment course by symptom domain, based on parent report. Note: RCADS = Revised Children’s Anxiety and Depression Scale. OCD = Obsessive–compulsive disorder.

An unconditional model examining change in parent-report RCADS Total T-scores indicated that youth affective symptoms improved significantly from pre- to mid-treatment (*B* = −0.55, *SE* = 0.11, *p* < .001, *d* = −0.54) and from mid- to post-treatment (*B* = −0.75, *SE* = 0.13, *p* < .001, *d* = −0.63). Parents reported significantly higher youth anxiety/depression symptoms than OCD symptoms (*B* = 5.83, *SE* = 0.71, *p* < .001, *d* = 0.47) and higher depression symptoms than anxiety symptoms (*B* = 1.65, *SE* = 0.82, *p* = .044, *d* = 0.12) at pre-treatment. Controlling for pre-treatment symptom severity and other covariates, OCD symptoms decreased at a slower rate than anxiety/depression symptoms (*B* = −0.37, *SE* = 0.15, *p* = .014, *d* = −0.14), and anxiety symptoms decreased at a slower rate than depression symptoms (*B* = −0.39, *SE* = 0.17, *p* = .026, *d* = −0.13) from pre- to mid-treatment. Simple slope analyses indicated that, while anxiety (*B* = −0.28, *SE* = 0.13, *p* = .026) and depression (*B* = −0.67, *SE* = 0.13, *p* < .001) symptoms both exhibited significant improvements from pre- to mid-treatment, OCD symptoms did not exhibit significant improvements during this time (*B* = −0.11, *SE* = 0.13, *p* = .400). On the contrary, there were no differences in rates of change between OCD and anxiety/depression from mid- to post-treatment (*B* = −0.02, *SE* = 0.18, *p* = .891, *d* = −0.01), nor were there differences in rates of change between anxiety and depression symptoms from mid- to post-treatment (*B* = 0.22, *SE* = 0.20, *p* = .269, *d* = 0.06). OCD symptoms improved significantly from mid- to post-treatment (*B* = −0.50, *SE* = 0.15, *p* < .001), and this rate of improvement was comparable to that of anxiety (*B* = −0.63, *SE* = 0.15, *p* < .001) and depression (*B* = −0.41, *SE* = 0.15 *p* = .006). See Table 5 for full multilevel model results, and see Figure 3 for trajectories of parent-reported youth symptoms across the UP-C/A treatment course.

### 3.3. Aim 2: Trajectories of Anxiety and Depression Symptoms Between Youth with and Without an OCD Diagnosis

#### 3.3.1. Youth Self-Reported Anxiety and Depression Symptoms

**Figure 4:**
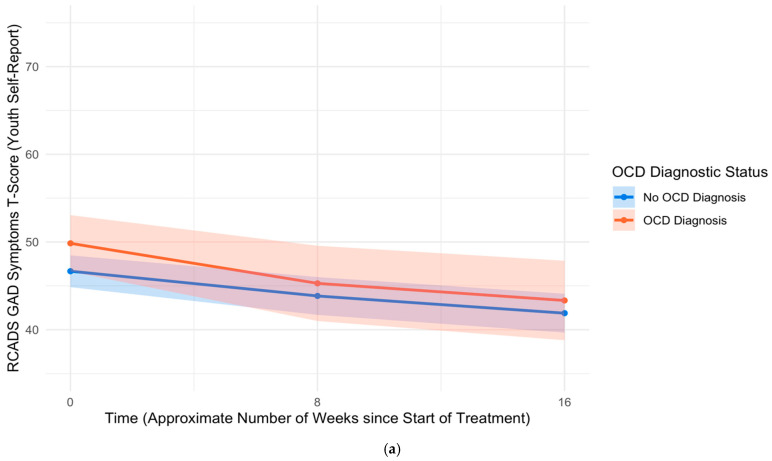

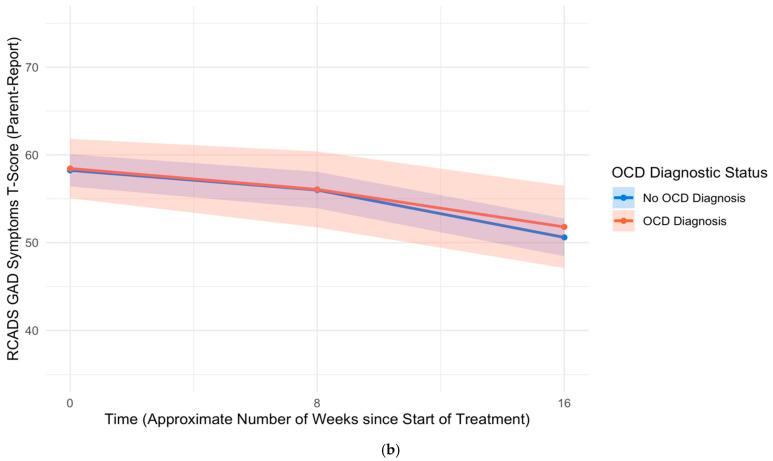
Trajectories of RCADS generalized anxiety symptom T-scores across treatment course, based on (**a**) youth self-report and (**b**) parent-report. Note: RCADS = Revised Children’s Anxiety and Depression Scale. GAD = Generalized anxiety disorder. OCD = Obsessive–compulsive disorder.

**Figure 5:**
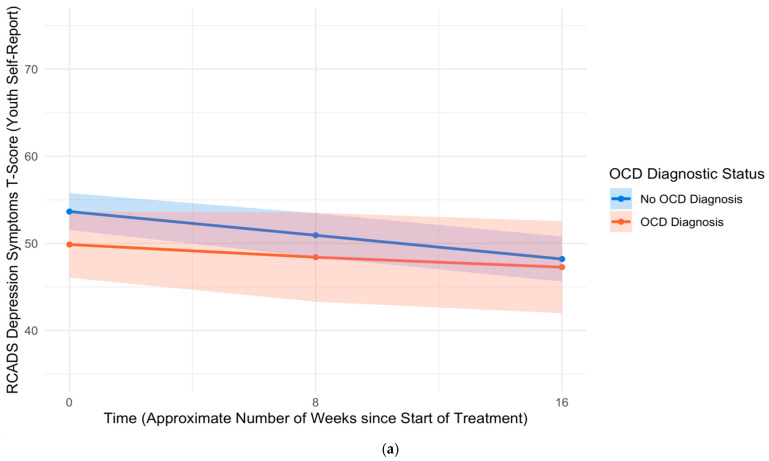

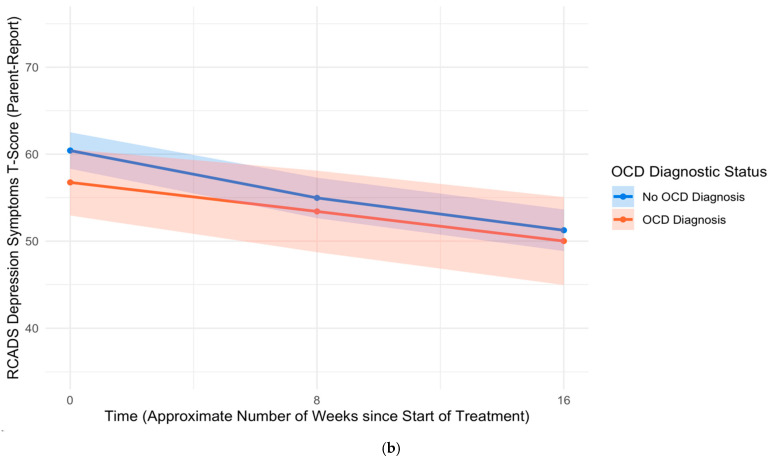
Trajectories of RCADS depression symptom T-scores across the treatment course, based on (**a**) youth self-report and (**b**) parent-report. Note: RCADS = Revised Children’s Anxiety and Depression Scale. OCD = Obsessive–compulsive disorder.

Youth with OCD reported higher levels of anxiety symptoms at pre-treatment than youth without OCD (*B* = 3.10, *SE* = 1.57, *p* = .049, *d* = 0.18). Controlling for pre-treatment differences in levels of anxiety symptoms and other covariates, there were no differences in rates of improvement in anxiety symptoms from pre- to mid-treatment (*B* = −0.23, *SE* = 0.32, *p* = .469, *d* = −0.07) or mid- to post-treatment (*B* < 0.01, *SE* = 0.36, *p* = .996, *d* < 0.01) between youth with and without OCD. Youth with OCD reported lower levels of depression symptoms at pre-treatment than youth without OCD (*B* = −3.73, *SE* = 1.82, *p* = .040, *d* = −0.19). Controlling for pre-treatment differences in levels of depression symptoms and other covariates, there were no differences in rates of improvement in depression symptoms from pre- to mid-treatment (*B* = 0.16, *SE* = 0.35, *p* = .645, *d* = 0.05) or mid- to post-treatment (*B* = 0.20, *SE* = 0.41, *p* = .627, *d* = 0.05) between youth with and without OCD. See Figure 4a and Figure 5a for trajectories of youth self-reported anxiety and depression symptoms, respectively, across the treatment course by OCD diagnostic status.

#### 3.3.2. Parent-Reported Anxiety and Depression Symptoms

Parents of youth with and without OCD reported similar levels of youth anxiety symptoms at pre-treatment (*B* = 0.36, *SE* = 1.66, *p* = .830, *d* = 0.02). Controlling for covariates, parents of youth with and without OCD did not report differences in rates of improvement of anxiety symptoms from pre- to mid-treatment (*B* = −0.02, *SE* = 0.32, *p* = .944, *d* = −0.01) or from mid- to post-treatment (*B* = 0.14, *SE* = 0.36, *p* = .699, *d* = 0.04). Parents of youth with and without OCD reported similar levels of youth depression symptoms at pre-treatment (*B* = −3.43, *SE* = 1.85, *p* = .064, *d* = −0.18). Controlling for covariates, there were no differences in rates of improvement in depression symptoms from pre- to mid-treatment (*B* = 0.26, *SE* = 0.31, *p* = .416, *d* = 0.09) or from mid- to post-treatment (*B* = 0.04, *SE* = 0.36, *p* = .919, *d* = 0.01) between the parents of youth with and without OCD. See Figure 4b and Figure 5b for trajectories of parent-reported anxiety and depression symptoms, respectively, across the treatment course by OCD diagnostic status.

### 3.4. Aim 3: Changes in Anxiety Sensitivity, Cognitive Flexibility, and Distress Tolerance Between Youth with and Without an OCD Diagnosis

#### 3.4.1. Aim 3a: Youth Self-Reported Anxiety Sensitivity

**Figure 6:**
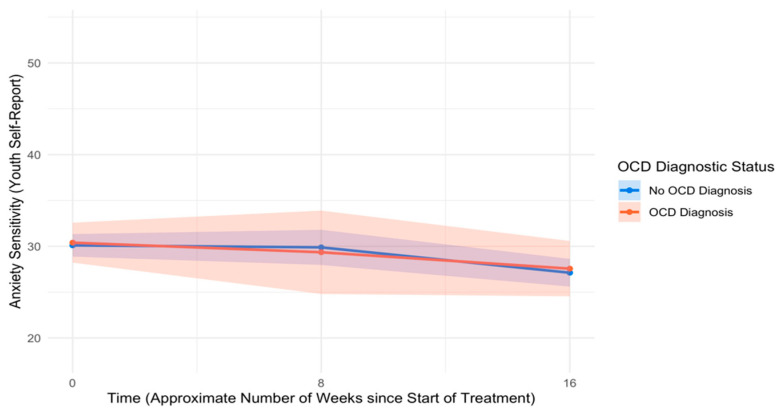
Trajectories of youth self-reported anxiety sensitivity across the treatment course. Note: OCD = Obsessive–compulsive disorder.

An unconditional model of change in anxiety sensitivity over time indicated that anxiety sensitivity did not change significantly from pre- to mid-treatment (*B* = −0.09, *SE* = 0.11, *p* = .407, *d* = −0.10) but improved significantly from mid- to post-treatment (*B* = −0.27, *SE* = 0.13, *p* = .031, *d* = −0.26). Controlling for covariates, youth with and without OCD reported similar levels of anxiety sensitivity at pre-treatment (*B* = 0.29, *SE* = 1.05, *p* = .782, *d* = 0.03) as well as similar rates of change in anxiety sensitivity from pre- to mid-treatment (*B* = −0.10, *SE* = 0.31, *p* = .735, *d* = −0.04) and mid- to post-treatment (*B* = 0.12, *SE* = 0.34, *p* = .715, *d* = 0.04). See Table 6 for full model results, and see Figure 6 for trajectories of anxiety sensitivity across the treatment course by OCD diagnostic status.

#### 3.4.2. Aim 3b: Youth Self-Reported Cognitive Flexibility

**Figure 7:**
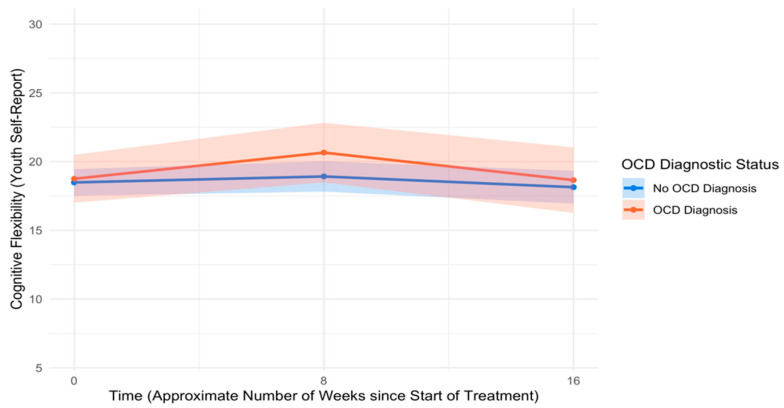
Trajectories of youth self-reported cognitive flexibility across the treatment course. Note: OCD = Obsessive–compulsive disorder.

An unconditional model of change in cognitive flexibility over time indicated no significant change in cognitive flexibility from pre- to mid-treatment (*B* = 0.04, *SE* = 0.05, *p* = .476, *d* = 0.07) or mid- to post-treatment (*B* = −0.10, *SE* = 0.06, *p* = .114, *d* = −0.16). Controlling for covariates, youth with and without OCD reported similar levels of cognitive flexibility at pre-treatment (*B* = 0.23, *SE* = 0.85, *p* = .783, *d* = 0.03) as well as similar rates of change in cognitive flexibility from pre- to mid-treatment (*B* = 0.18, *SE* = 0.15, *p* = .213, *d* = 0.13) and mid- to post-treatment (*B* = −0.16, *SE* = 0.17, *p* = .371, *d* = −0.10). See Table 7 for full model results, and see Figure 7 for trajectories of cognitive flexibility across the treatment course by OCD diagnostic status.

#### 3.4.3. Aim 3c: Youth Self-Reported Distress Tolerance

**Figure 8:**
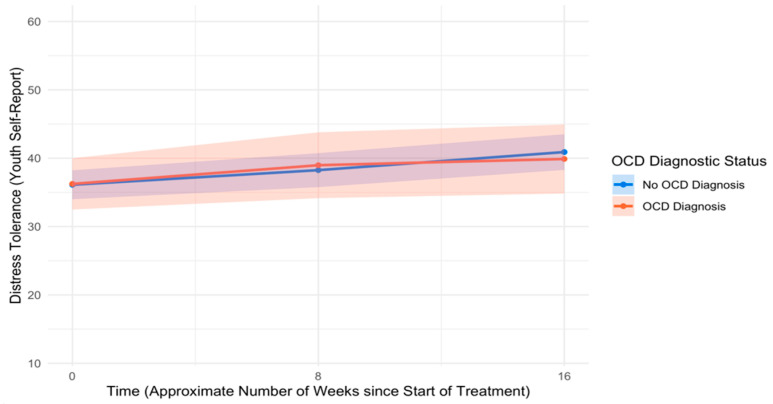
Trajectories of youth self-reported distress tolerance across the treatment course. Note: OCD = Obsessive–compulsive disorder.

An unconditional model of change in distress tolerance over time indicated that distress tolerance improved significantly from pre- to mid-treatment (*B* = 0.34, *SE* = 0.11, *p* = .002, *d* = 0.33) and from mid- to post-treatment (*B* = 0.28, *SE* = 0.13, *p* = .031, *d* = 0.24). Controlling for covariates, youth with and without OCD reported similar levels of distress tolerance at pre-treatment (*B* = 0.10, *SE* = 1.81, *p* = .958, *d* = 0.01) as well as similar rates of change in distress tolerance from pre- to mid-treatment (*B* = 0.07, *SE* = 0.32, *p* = .828, *d* = 0.02) and mid- to post-treatment (*B* = −0.22, *SE* = 0.38, *p* = .565, *d* = −0.07). See Table 8 for full model results, and see Figure 8 for trajectories of distress tolerance across the treatment course by OCD diagnostic status.

## 4. Discussion

To our knowledge, this is the first study to compare trajectories of OCD, anxiety, and depression symptoms among a large, transdiagnostic sample of youth receiving UP-C/A treatment. Overall, our findings indicated that OCD symptoms improved significantly during treatment, and youth with and without an OCD diagnosis exhibited comparable improvements in anxiety, depression, and transdiagnostic treatment targets (i.e., anxiety sensitivity, cognitive flexibility, and distress tolerance). However, trajectories of OCD symptom improvement differed from trajectories of anxiety and depression symptom improvement, pointing to areas for potential treatment adaptation to maximize clinical benefit for youth with OCD.

Our first aim was to compare trajectories of OCD, anxiety, and depression symptoms during UP-C/A treatment. Anxiety and depression symptoms significantly improved during the first half of UP-C/A treatment, while OCD symptoms showed no significant change until the second half of treatment. During the latter half of treatment, OCD symptoms improved significantly and at rates comparable to those of anxiety and depression, with consistent patterns across youth and parent reports. These findings suggest that the psychoeducational and cognitive-focused content emphasized early in UP-C/A treatment may be less effective for reducing OCD symptoms compared to the exposure-focused content presented in the second half of a typical UP-C/A treatment course. While the UP-C/A is designed to be modular and flexibly tailored to a range of symptom domains, less experienced clinicians may more strictly adhere to sequential ordering and general, client non-specific examples. Our data suggest that to enhance outcomes for youth with OCD, clinicians should consider introducing exposure-focused modules earlier and embedding exposure language throughout UP-C/A treatment. For example, “opposite action” in UP-C/A Module 3 can be and is often reframed as an exposure strategy rather than solely behavioral activation. Still, as this study did not directly assess the timing or dose of UP-C/A intervention content delivered, it is also possible that OCD symptoms simply require more time to exhibit change compared to anxiety and depression symptoms. Moreover, there may be lagged effects of specific UP-C/A content on symptom change over time (i.e., content presented earlier in treatment may not exert its effect until later in treatment), and such lagged effects could hypothetically differ by symptom domain. Future studies examining how OCD and other symptom domains respond to specific UP-C/A modules are needed to inform efforts to tailor the intervention depending on clients’ presenting needs.

In addition to assessing OCD symptoms dimensionally, we further examined whether youth with and without an OCD diagnosis exhibited comparable treatment gains. Compared to youth without OCD, youth with OCD reported higher self-reported (but not parent-reported) anxiety symptoms and lower self-reported (but not parent-reported) depression symptoms at pre-treatment. The latter finding contradicts existing literature suggesting that OCD is commonly associated with heightened depression symptoms and can itself incur risk for later depression [84,85]. This finding may be explained by diagnostic overshadowing (i.e., the tendency for one significant presenting concern to eclipse other, less prominent symptoms) or by differences in self-stigma and/or self-reporting tendencies among youth with and without OCD [86]. In any case, controlling for differences in pre-treatment symptoms, youth with and without OCD exhibited comparable improvements in anxiety and depression symptoms across the treatment course, suggesting that the presence of an OCD diagnosis does not inhibit the efficacy of the UP-C/A for other co-occurring affective disorders. This finding contributes to a growing body of literature highlighting the benefit of transdiagnostic treatments over single domain protocols for youth presenting with multiple co-occurring psychiatric disorders [40,41,42].

Our third and final aim was to assess whether UP-C/A treatment elicits change in its intended transdiagnostic mechanisms to the same extent for youth with and without OCD. Youth with and without OCD exhibited comparable levels of anxiety sensitivity, cognitive flexibility, and distress tolerance at pre-treatment, supporting existing theories highlighting each of these factors as higher-order, domain non-specific processes implicated in a range of youth affective disorders [46,47,48]. Youth with and without OCD exhibited comparable rates of improvement in self-reported anxiety sensitivity and distress tolerance. However, youth self-reported cognitive flexibility did not exhibit significant change during treatment in the sample of youth with OCD nor in the sample of youth without OCD. This apparent lack of change is inconsistent with other studies suggesting that the cognitive-focused UP-C/A content does tap its intended mechanism [87] and indicates possible issues with the measurement of this construct in this study. It is possible that some of the items included in the ERQ-CA measure [73] are not aligned with the conceptualization of cognitive flexibility in the UP-C/A. For example, the ERQ-CA cognitive flexibility/reappraisal subscale includes items such as “When I want to feel less bad (e.g., sad, angry or worried), I think about something different”, which could be interpreted as avoidance rather than a cognitive reappraisal process. The desired outcome of the cognitive-focused UP-C/A content is not to avoid negative thoughts but rather to recognize and accept negative, positive, and neutral thoughts. Further research is needed to refine the measurement of cognitive flexibility during the UP-C/A and similar treatments. That notwithstanding, our findings indicate that the UP-C/A taps at least two out of three of its intended mechanisms for youth with OCD, further supporting the applicability of this transdiagnostic, emotion-focused treatment for this clinical population.

This study has several limitations that warrant consideration. First, certain characteristics of the participant sample may limit the generalizability of the study findings to other youth samples. Youth participants in this study were predominantly White and Hispanic/Latino/a, which is consistent with the demographic characteristics of youth presenting to the recruiting clinic but does not represent the larger population of youth seeking treatment for OCD and affective disorders in the United States. While we consider the clinical heterogeneity of our participant sample to be a strength of this study, only a minority of youth participants in this sample were diagnosed with OCD, and it is possible that the relatively small sample of youth with OCD limited our ability to detect small effect size differences between groups of youth with and without OCD.

In addition to the limitations related to the characteristics of our sample, there are limitations related to the measurement of constructs examined in this study. While the RCADS has been used to assess change in OCD symptoms in other youth treatment research [61], this measure captures only a limited range of OCD symptoms. OCD is a highly heterogeneous disorder encompassing a wide range of topographical symptoms, and it is possible that the items included in the RCADS OCD subscale did not resonate with all youth with OCD in this study. Previous literature indicates that brief measures designed to assess a broad range of psychiatric symptoms may underestimate treatment effects compared to more specialized, disorder-specific measures [88]. OCD-specific measures such as the Children’s Yale–Brown Obsessive–Compulsive Scale (CY-BOCS) [89] may allow for a more nuanced assessment of OCD symptoms and change over time. In this vein, it is noteworthy that our sample of youth receiving treatment for affective disorders appeared to exhibit overall subclinical levels of affective symptoms (including anxiety, depression, and OCD symptoms) as indicated by the RCADS youth and parent reports. The levels of symptoms reported in this study are comparable to those reported in similar previous studies of youth receiving UP-C/A treatment [90] and are likely explained by the transdiagnostic nature of the sample and the measure (i.e., not all youth should endorse all items). It is also important to note that we did not examine parent reports of the transdiagnostic emotion dysfunctions assessed in this study, nor did we examine parent-related UP-C/A treatment targets (e.g., reduction in parental accommodation). Examining parent reports of youth emotion dysfunctions and of their own emotional responding to their children’s symptoms could provide additional information to characterize mechanism changes in youth with and without OCD during UP-C/A treatment.

We did not directly assess clinicians’ use and ordering of UP-C/A modules in this study. While we examined symptom trajectories from pre- to mid-treatment and mid- to post-treatment separately as to approximate the point at which exposure-based strategies are often introduced during UP-C/A treatment (i.e., around session 8 if delivering content in a sequential order), it is unlikely that all youth included in this study received the same modules in the same order. Therefore, we are unable to draw firm conclusions about the relationship between symptom trajectories during the examined time frames and particular treatment strategies, such as exposure. It is possible that youth with OCD received a greater dose of exposure content and/or began exposure earlier in treatment than did youth without OCD given the evident importance of exposure for this symptom presentation. Even so, our findings suggest that, while OCD symptoms may require a slightly longer treatment duration and/or an earlier introduction of exposure-based strategies to exhibit change, youth with OCD benefit comparably to those without OCD from UP-C/A treatment.

Our findings contribute to efforts to expand the range of evidence-based approaches for youth with OCD and particularly for those with other co-occurring affective disorders. Future research should assess the impact of individual UP-C/A modules on specific symptoms to inform intervention tailoring. On the other hand, too much tailoring based on diagnostic categories may inadvertently undermine the transdiagnostic principles that form the foundation of the intervention. Assessing the impacts of individual UP-C/A modules on specific emotion regulation processes and skills may more closely align with the theoretical underpinnings of the intervention. Research conducted with the adult UP indicates that some UP modules elicit skill-specific changes (e.g., the cognitive flexibility module leads to changes in cognitive flexibility), while other UP modules lead to broader, cross-skill improvements (e.g., the “countering emotional behaviors” module, including exposure, leads to change across multiple skills) [91]. If the same is true for youth, it may be worthwhile to consider the potential benefit of presenting the more broadly applicable UP-C/A modules earlier in the treatment course and saving the skill-specific modules for when and for whom they are needed. In the case of exposure, however, clinicians often report reluctance to commence exposure early in treatment due to concern that the client may be too impaired by symptoms to engage in exposure and/or worry that starting exposure too soon could hinder clinician–client rapport [35]. Researchers aiming to determine the optimal order and sequencing of UP-C/A modules to maximize efficiency and effectiveness should supplement studies of UP-C/A module effects with qualitative research assessing how clinicians in routine practice make decisions about the ordering and prioritization of CBT treatment content. For youth with OCD and other related disorders for which exposure is the key driver of symptom change, it would be especially important to assess what factors signify to clinicians that a client is ready to engage in exposure (e.g., whether there are certain skills that must be evidenced prior to starting exposure).

A direct comparison of UP-C/A to ERP would allow for a more rigorous assessment of the efficacy and effect sizes of the UP-C/A as compared to the front-line intervention for OCD. While treatment using the UP-C/A may more explicitly target co-occurring symptoms, traditional ERP for OCD may also exhibit downstream effects on co-occurring symptoms. For example, ERP decreases avoidance and increases approach behavior [92], which could spur change in co-occurring anxiety and depression symptoms [93,94]. Assessing the comparative efficacy of the UP-C/A and ERP for OCD symptoms as well as a range of commonly co-occurring symptoms (e.g., anxiety, depression) and transdiagnostic treatment targets (e.g., anxiety sensitivity, cognitive flexibility, and distress tolerance) would aid in the determination of which treatment is best for whom.

## Figures and Tables

**Table 1:** Sample characteristics.

*M* (*SD*) or %	Full Sample (*N* = 388)	Youth with an OCD Diagnosis (*n* = 60)	Youth Without an OCD Diagnosis (*n* = 328)
Age	12.5 (2.9)	12.7 (2.8)	12.5 (2.9)
Biological sex	51.5% Female	70.0% Female	48.0% Female
	48.5% Male	30.0% Male	51.0% Male
Race	90.1% White	93.2% White	89.5% White
	2.1% Black	1.7% Black	2.2% Black
	3.1% Asian	5.1% Asian	2.8% Asian
	0.3% American Indian or Alaska Native	0.% American Indian or Alaska Native	0.2% American Indian or Alaska Native
	2.3% Multiracial	0.0% Multiracial	2.8% Multiracial
	2.1% Other	0.0% Other	2.5% Other
Ethnicity	70.8% Hispanic/Latino/a	75.0% Hispanic/Latino/a	69.1% Hispanic/Latino/a
	29.2% Non-Hispanic/Latino/a	25.0% Non-Hispanic/Latino/a	30.9% Non-Hispanic/Latino/a
Income	USD 139,051 (USD 153,972)	USD 161,535 (USD 136,790)	USD 134,922 (USD 156,776)
Any anxiety disorder	96.4% Has an anxiety disorder	83.3% Has an anxiety disorder	98.8% Has an anxiety disorder
	3.6% Does not have an anxiety disorder	16.7% Does not have an anxiety disorder	1.2% Does not have an anxiety disorder
Any depressive disorder	35.8% Has a depressive disorder	25.0% Has a depressive disorder	37.8% Has a depressive disorder
	64.2% Does not have a depressive disorder	75.0% Does not have a depressive disorder	62.2% Does not have a depressive disorder
Baseline symptom severity (RCADS Total T-score Youth Self-Report)	52.2 (15.6)	59.4 (16.7)	51.1 (15.1)
Baseline symptom severity (RCADS Total T-score Parent-Report)	62.4 (14.6)	67.0 (16.9)	61.6 (14.0)
Number of diagnoses	2.9 (1.2)	3.3 (1.3)	2.9 (1.1)
Treatment manual	51.3% UP-C	53.3% UP-C	47.9% UP-C
	48.7% UP-A	46.7% UP-A	52.1% UP-A
Treatment format	78.9% Individual	91.7% Individual	76.5% Individual
	21.1% Group	8.3% Group	23.5% Group
Treatment location	55.2% In-person	38.3% In-person	58.2% In-person
	44.8% Telehealth/hybrid	61.7% Telehealth/hybrid	41.8% Telehealth/hybrid
Number of treatment sessions attended	15.0 (7.5)	17.9 (8.6)	14.4 (7.1)

Note: *M* = Mean. *SD* = Standard deviation. RCADS = Revised Children’s Anxiety and Depression Scale. UP-C = Unified Protocol for Transdiagnostic Treatment of Emotional Disorders in Children. UP-A = Unified Protocol for Transdiagnostic Treatment of Emotional Disorders in Adolescents.

**Table 2:** UP-C/A treatment modules, theorized mechanisms of change, and approximate treatment schedule.

UP-C “CLUES” Skill and UP-A Module	Treatment Content Description	Theorized Mechanisms of Change	Approximate Session Number When Delivered in Sequential Order
UP-C ‘C’ Skill: *Consider How I Feel* UP-A Module 1: *Building and Keeping Motivation*	Discussion of treatment goals and assessment of barriers to treatment	Foster treatment motivation and therapeutic alliance	Session 1
UP-C ‘C’ Skill: *Consider How I Feel* UP-A Module 2: *Getting to Know Your Emotions and Behaviors*	Psychoeducation about the nature and purpose of emotions, three parts of emotions, and cycle of emotional behaviors	Increase emotional awareness	Sessions 1–2
UP-C ‘C’ Skill: *Consider How I Feel* UP-A Module 3: *Introduction to Emotion-Focused Behavioral Experiments*	Behavioral activation and other “opposite actions” for emotional behaviors	Decrease avoidance and other maladaptive reactions to strong emotions	Sessions 2–3
UP-C ‘C’ Skill: *Consider How I Feel* UP-A Module 4: *Awareness of Physical Sensations*	Psychoeducation about physiological symptoms, body scanning, and interoceptive exposure	Increase tolerance of physiological symptoms related to emotions and improve anxiety sensitivity	Session 4
UP-C ‘L’ Skill: *Look at My Thoughts* UP-C ‘U’ Skill: *Use Detective Thinking and Problem Solving* UP-A Module 5: *Being Flexible in Your Thinking*	Identification of cognitive distortions, practice cognitive reappraisal, and problem solving	Increase cognitive flexibility	Sessions 5–7
UP-C ‘E’ Skill: *Experience My Emotions* UP-A Module 6: *Awareness of Emotional Experiences*	Mindfulness, including present-moment awareness and non-judgmental awareness exercises	Increase cognitive flexibility and improve distress tolerance	Session 8
UP-C ‘E’ Skill: *Experience My Emotions* UP-A Module 7: *Situational Emotion Exposures*	Imaginal and in vivo exposure to emotional triggers	Reduce anxiety sensitivity and improve distress tolerance	Sessions 9–14
UP-C ‘S’ Skill: *Stay Happy and Healthy* UP-A Module 8: *Keeping It Going, Maintaining Your Gains*	Skill review and celebration of progress	Relapse prevention	Sessions 15–16

Note: UP-C = Unified Protocol for Transdiagnostic Treatment of Emotional Disorders in Children. UP-A = Unified Protocol for Transdiagnostic Treatment of Emotional Disorders in Adolescents.

**Table 3:** Skewness, kurtosis, and bivariate correlations among outcome measures.

Measure	Skewness	Kurtosis	1	2	3	4	5	6	7	8	9	10
1. Baseline symptom severity (RCADS Total T-score, Youth Self-Report)	0.73	0.52										
2. Baseline symptom severity (RCADS Total T-score, Parent-Report)	0.71	0.87	0.22 **									
3. OCD symptoms (RCADS OCD T-score, Youth Self-Report)	1.17	1.48	0.77 **	0.15 *								
4. OCD symptoms (RCADS OCD T-score, Parent-Report)	1.81	3.76	0.16 **	0.66 **	0.28 **							
5. Anxiety symptoms (RCADS GAD T-score, Youth Self-Report)	0.43	−0.55	0.83 **	0.21 **	0.66 **	0.20 **						
6. Anxiety symptoms (RCADS GAD T-score, Parent-Report)	0.75	0.21	0.13 *	0.80 **	0.10	0.55 **	0.24 **					
7. Depression symptoms (RCADS Depression T-score, Youth Self-Report)	0.88	0.37	0.84 **	0.18 **	0.52 **	0.03	0.61 **	0.06				
8. Depression symptoms (RCADS Depression T-score, Parent-Report)	0.88	0.85	0.31 **	0.69 **	0.14 *	0.26 **	0.20 **	0.42 **	0.43 **			
9. Anxiety sensitivity (CASI Total Score, Youth Self-Report)	0.63	−0.11	0.74 **	0.21 **	0.61 **	0.15 *	0.58 **	0.13 *	0.59 **	0.21 **		
10. Cognitive flexibility (ERQ-CA Reappraisal Score, Youth Self-Report)	−0.18	−0.07	−0.03	0.07	0.06	0.06	−0.01	0.01	−0.12 *	−0.01	0.18 **	
11. Distress tolerance (DTS GDT Score, Youth Self-Report)	0.24	−0.55	−0.49 **	−0.21 **	−0.43 **	−0.17 **	−0.44 **	−0.21 **	−0.39 **	−0.18 **	−0.51 **	0.05

* *p* < .05, ** *p* < .01. Note: RCADS = Revised Children’s Anxiety and Depression Scale. OCD = Obsessive–compulsive disorder. GAD = Generalized anxiety disorder. CASI = Childhood Anxiety Sensitivity Index. ERQ-CA = Emotion Regulation Questionnaire for Children and Adolescents. DTS-GDT = Distress Tolerance Scale–Global Distress Tolerance.

**Table 4:** Multilevel model of change in youth-reported symptoms across the treatment course.

Predictor of RCADS T-Score, Youth Self-Report	*B*	*SE*	*t*	*p*	95% CI	Cohen’s *d*	VIF	Tol
Intercept	16.53	2.26	7.32	<.001 ***	(12.11, 20.96)	−−	−−	−−
Time (Weeks 0 to 8)	−0.28	0.09	−3.05	.002 **	(−0.46, −0.10)	−0.30	1.54	0.65
Time (Weeks 8 to 16)	−0.30	0.11	−2.80	.005 **	(−0.51, −0.09)	−0.28	1.51	0.66
Age	−0.06	0.15	−0.39	.694	(−0.35, 0.23)	−0.04	1.42	0.70
Biological sex (0 = Male)	−0.11	0.72	−0.15	.878	(−1.52, 1.30)	−0.02	1.07	0.93
Race (0 = White)	−1.04	1.23	−0.84	.398	(−3.45, 1.37)	−0.10	1.11	0.90
Ethnicity (0 = Non-Hispanic/Latino/a)	1.10	0.79	1.39	.163	(−0.45, 2.65)	0.16	1.18	0.85
Family income (Z-scored)	−0.84	0.35	−2.38	.017 *	(−1.54, −0.15)	−0.26	1.16	0.86
Baseline symptom severity	0.63	0.03	24.46	<.001 ***	(0.58, 0.68)	2.79	1.26	0.80
Number of diagnoses	0.08	0.33	0.24	.813	(−0.56, 0.72)	0.03	1.12	0.89
Treatment format (0 = Individual)	−0.68	0.98	−0.70	.485	(−2.61, 1.24)	−0.08	1.27	0.79
Treatment location (0 = In-person)	1.17	0.75	1.56	.119	(−0.30, 2.65)	0.18	1.10	0.91
Number of treatment sessions attended	<0.01	0.05	−0.03	.972	(−0.10, 0.10)	<0.01	1.09	0.92
Symptom domain (OCD versus Anxiety/Depression)	4.50	0.63	7.17	<.001 ***	(3.27, 5.73)	0.44	1.86	0.54
Symptom domain (Anxiety versus Depression)	3.65	0.73	4.97	<.001 ***	(2.21, 5.09)	0.31	1.86	0.54
Time (Weeks 0 to 8) × Symptom domain (OCD versus Anxiety/Depression)	−0.28	0.14	−2.00	.046 *	(−0.55, −0.01)	−0.12	2.81	0.36
Time (Weeks 0 to 8) × Symptom domain (Anxiety versus Depression)	0.01	0.16	0.06	.950	(−0.30, 0.32)	<0.01	2.81	0.36
Time (Weeks 8 to 16) × Symptom domain (OCD versus Anxiety/Depression)	0.06	0.16	0.39	.695	(−0.26, 0.38)	0.02	1.95	0.51
Time (Weeks 8 to 16) × Symptom domain (Anxiety versus Depression)	−0.06	0.19	−0.30	.768	(−0.42, 0.31)	−0.02	1.95	0.51

Note: The dependent variable is the RCADS Youth Self-Report Total T-score. RCADS = Revised Children’s Anxiety and Depression Scale. SE = Standard error. CI = Confidence interval. VIF = Variance inflation factor. Tol = Tolerance. OCD = Obsessive–compulsive disorder. * *p* < .05, ** *p* < .01, *** *p* < .001.

**Table 5:** Multilevel model of change in parent-reported symptoms across the treatment course.

Predictor of RCADS T-Score, Parent-Report	*B*	*SE*	*t*	*p*	95% CI	Cohen’s *d*	VIF	Tol
Intercept	21.48	2.51	8.55	<.001 ***	(16.55, 26.40)	−−	−−	−−
Time (Weeks 0 to 8)	−0.35	0.08	−4.56	<.001 ***	(−0.50, −0.20)	−0.44	1.45	0.69
Time (Weeks 8 to 16)	−0.51	0.09	−5.66	<.001 ***	(−0.69, −0.34)	−0.54	1.46	0.69
Age	−0.03	0.14	−0.21	.830	(−0.30, 0.24)	−0.02	1.38	0.73
Biological sex (0 = Male)	0.63	0.66	0.95	.340	(−0.66, 1.93)	0.11	1.06	0.94
Race (0 = White)	0.13	1.13	0.11	.909	(−2.09, 2.35)	0.01	1.10	0.91
Ethnicity (0 = Non-Hispanic/Latino/a)	0.35	0.75	0.47	.642	(−1.12, 1.81)	0.05	1.17	0.86
Family income (Z-scored)	−0.33	0.34	−0.96	.337	(−1.00, 0.34)	−0.11	1.17	0.85
Baseline symptom severity	0.53	0.02	23.20	<.001 ***	(0.49, 0.58)	2.63	1.07	0.93
Number of diagnoses	0.75	0.30	2.52	.012 *	(0.17, 1.33)	0.28	1.11	0.90
Treatment format (0 = Individual)	−1.14	0.89	−1.28	.201	(−2.90, 0.61)	−0.15	1.24	0.81
Treatment location (0 = In-person)	0.63	0.68	0.92	.360	(−0.72, 1.97)	0.10	1.10	0.91
Number of treatment sessions attended	0.05	0.05	0.98	.329	(−0.05, 0.14)	0.10	1.06	0.94
Symptom domain (OCD versus Anxiety/Depression)	5.83	0.71	8.24	<.001 ***	(4.44, 7.22)	0.47	1.99	0.50
Symptom domain (Anxiety versus Depression)	1.65	0.82	2.01	.044 *	(0.04, 3.26)	0.12	1.99	0.50
Time (Weeks 0 to 8) × Symptom domain (OCD versus Anxiety/Depression)	−0.37	0.15	−2.47	.014 *	(−0.66, −0.08)	−0.14	2.88	0.35
Time (Weeks 0 to 8) × Symptom domain (Anxiety versus Depression)	−0.39	0.17	−2.23	.026 *	(−0.73, −0.05)	−0.13	2.88	0.35
Time (Weeks 8 to 16) × Symptom domain (OCD versus Anxiety/Depression)	−0.02	0.18	−0.14	.891	(−0.37, 0.32)	−0.01	1.88	0.53
Time (Weeks 8 to 16) × Symptom domain (Anxiety versus Depression)	0.22	0.20	1.11	.269	(−0.17, 0.62)	0.06	1.88	0.53

Note: The dependent variable is the RCADS Parent-Report Total T-score. RCADS = Revised Children’s Anxiety and Depression Scale. SE = Standard error. CI = Confidence interval. VIF = Variance inflation factor. Tol = Tolerance. OCD = Obsessive–compulsive disorder. * *p* < .05, *** *p* < .001.

**Table 6:** Multilevel model of change in anxiety sensitivity across the treatment course.

Predictor of CASI Score	*B*	*SE*	*t*	*p*	95% CI	Cohen’s *d*	VIF	Tol
Intercept	13.84	2.05	6.76	<.001 ***	(9.82, 17.87)	−−	−−	−−
Time (Weeks 0 to 8)	−0.03	0.11	−0.24	.810	(−0.25, 0.19)	−0.03	3.08	0.32
Time (Weeks 8 to 16)	−0.35	0.12	−2.79	.006 **	(−0.59, −0.10)	−0.32	3.14	0.32
Age	0.09	0.13	0.64	.523	(−0.18, 0.35)	0.07	1.44	0.70
Biological sex (0 = Male)	1.93	0.66	2.95	.003 **	(0.65, 3.22)	0.35	1.11	0.90
Race (0 = White)	0.68	1.10	0.62	.538	(−1.49, 2.85)	0.07	1.12	0.89
Ethnicity (0 = Non-Hispanic/Latino/a)	0.86	0.72	1.20	.232	(−0.55, 2.27)	0.14	1.19	0.84
Family income (Z-scored)	−0.12	0.31	−0.38	.705	(−0.73, 0.50)	−0.05	1.17	0.85
Baseline symptom severity	0.27	0.02	11.78	<.001 ***	(0.22, 0.31)	1.36	1.28	0.78
Number of diagnoses	0.17	0.29	0.57	.569	(−0.41, 0.74)	0.07	1.14	0.88
Treatment format (0 = Individual)	−0.87	0.89	−0.97	.331	(−2.63, 0.89)	−0.11	1.28	0.78
Treatment location (0 = In-person)	−0.34	0.67	−0.50	.615	(−1.66, 0.98)	−0.06	1.15	0.87
Number of treatment sessions attended	−0.03	0.04	−0.62	.539	(−0.12, 0.06)	−0.07	1.10	0.91
OCD diagnosis (0 = No)	0.29	1.05	0.28	.782	(−1.78, 2.36)	0.03	1.40	0.71
Time (Weeks 0 to 8) × OCD diagnosis	−0.10	0.31	−0.34	.735	(−0.71, 0.50)	−0.04	4.06	0.25
Time (Weeks 8 to 16) × OCD diagnosis	0.12	0.34	0.37	.715	(−0.54, 0.78)	0.04	3.80	0.26

Note: The dependent variable is the CASI total score. CASI = Childhood Anxiety Sensitivity Index. SE = Standard error. CI = Confidence interval. VIF = Variance inflation factor. Tol = Tolerance. OCD = Obsessive–compulsive disorder. ** *p *< .01, *** *p* < .001.

**Table 7:** Multilevel model of change in cognitive flexibility across the treatment course.

Predictor of ERQ-CA Score	*B*	*SE*	*t*	*p*	95% CI	Cohen’s *d*	VIF	Tol
Intercept	17.88	1.62	11.02	<.001 ***	(14.69, 21.06)	−−	−−	−−
Time (Weeks 0 to 8)	0.06	0.05	1.02	.306	(−0.05, 0.16)	0.11	1.59	0.63
Time (Weeks 8 to 16)	−0.10	0.07	−1.45	.148	(−0.23, 0.03)	−0.16	1.57	0.64
Age	0.09	0.11	0.81	.420	(−0.12, 0.29)	0.10	1.43	0.70
Biological sex (0 = Male)	0.14	0.52	0.26	.793	(−0.88, 1.15)	0.03	1.11	0.90
Race (0 = White)	0.53	0.88	0.61	.542	(−1.19, 2.26)	0.08	1.13	0.89
Ethnicity (0 = Non-Hispanic/Latino/a)	1.31	0.57	2.30	.022 *	(0.19, 2.44)	0.29	1.20	0.83
Family income (Z-scored)	0.10	0.25	0.39	.696	(−0.40, 0.60)	0.05	1.17	0.86
Baseline symptom severity	−0.02	0.02	−1.23	.220	(−0.06, 0.01)	−0.15	1.29	0.77
Number of diagnoses	0.11	0.23	0.46	.646	(−0.35, 0.56)	0.06	1.13	0.89
Treatment format (0 = Individual)	<0.01	0.69	<0.01	.999	(−1.37, 1.36)	<0.01	1.28	0.78
Treatment location (0 = In-person)	−0.76	0.53	−1.41	.158	(−1.81, 0.29)	−0.17	1.12	0.89
Number of treatment sessions attended	−0.02	0.04	−0.46	.642	(−0.09, 0.05)	−0.05	1.10	0.91
OCD diagnosis (0 = No)	0.23	0.85	0.28	.783	(−1.43, 1.90)	0.03	1.48	0.68
Time (Weeks 0 to 8) × OCD diagnosis	0.18	0.15	1.25	.213	(−0.10, 0.47)	0.13	2.09	0.48
Time (Weeks 8 to 16) × OCD diagnosis	−0.16	0.17	−0.89	.371	(−0.50, 0.19)	−0.10	1.74	0.57

Note: The dependent variable is the ERQ-CA total score. ERQ-CA = Emotion Regulation Questionnaire for Children and Adolescents. SE = Standard error. CI = Confidence interval. VIF = Variance inflation factor. Tol = Tolerance. OCD = Obsessive–compulsive disorder. * *p* < .05, *** *p* < .001.

**Table 8:** Multilevel model of change in distress tolerance across the treatment course.

Predictor of DTS-GDT Score	*B*	*SE*	*t*	*p*	95% CI	Cohen’s *d*	VIF	Tol
Intercept	51.76	3.49	14.83	<.001 ***	(44.90, 58.62)	−−	−−	−−
Time (Weeks 0 to 8)	0.27	0.12	2.18	.029 *	(0.03, 0.51)	0.24	1.69	0.59
Time (Weeks 8 to 16)	0.33	0.15	2.25	.025 *	(0.04, 0.62)	0.26	1.68	0.60
Age	0.17	0.23	0.72	.471	(−0.28, 0.62)	0.09	1.42	0.70
Biological sex (0 = Male)	−1.87	1.12	−1.67	.095	(−4.07, 0.32)	−0.20	1.10	0.91
Race (0 = White)	0.67	1.89	0.36	.722	(−3.04, 4.39)	0.04	1.12	0.89
Ethnicity (0 = Non-Hispanic/Latino/a)	−1.12	1.22	−0.91	.362	(−3.52, 1.29)	−0.11	1.18	0.85
Family income (Z-scored)	0.53	0.55	0.95	.341	(−0.56, 1.61)	0.12	1.17	0.86
Baseline symptom severity	−0.28	0.04	−7.06	<.001 ***	(−0.36, −0.20)	−0.84	1.28	0.78
Number of diagnoses	−0.18	0.52	−0.36	.720	(−1.20, 0.83)	−0.04	1.14	0.88
Treatment format (0 = Individual)	0.94	1.53	0.61	.539	(−2.07, 3.95)	0.07	1.26	0.79
Treatment location (0 = In-person)	−1.73	1.15	−1.50	.133	(−4.00, 0.53)	−0.18	1.10	0.91
Number of treatment sessions attended	−0.07	0.08	−0.85	.393	(−0.22, 0.09)	−0.10	1.10	0.91
OCD diagnosis (0 = No)	0.10	1.81	0.05	.958	(−3.47, 3.66)	0.01	1.44	0.69
Time (Weeks 0 to 8) × OCD diagnosis	0.07	0.32	0.22	.828	(−0.56, 0.70)	0.02	2.18	0.46
Time (Weeks 8 to 16) × OCD diagnosis	−0.22	0.38	−0.58	.565	(−0.96, 0.53)	−0.07	1.86	0.54

Note: The dependent variable is the DTS-GDT score. DTS-GDT = Distress Tolerance Scale–Global Distress Tolerance. SE = Standard error. CI = Confidence interval. VIF = Variance inflation factor. Tol = Tolerance. OCD = Obsessive–compulsive disorder. * *p* < .05, *** *p* < .001.

## Data Availability

The data included in this study are not currently available due to privacy restrictions but may be made available upon reasonable request to the corresponding author.
